# Expressed alleles of imprinted IGF2, DLK1 and MEG3 colocalize in 3D-preserved nuclei of porcine fetal cells

**DOI:** 10.1186/s12860-016-0113-9

**Published:** 2016-10-01

**Authors:** Yvette Lahbib-Mansais, Harmonie Barasc, Maria Marti-Marimon, Florence Mompart, Eddie Iannuccelli, David Robelin, Juliette Riquet, Martine Yerle-Bouissou

**Affiliations:** GenPhySE, Université de Toulouse, INRA, INPT, ENVT, Castanet Tolosan, France

**Keywords:** Fetal pig, Imprinting, IGF2, DLK1/MEG3, 3D RNA-DNA FISH, Interchromosomal associations, Colocalization, Muscle, Liver

## Abstract

**Background:**

To explore the relationship between spatial genome organization and gene expression in the interphase nucleus, we used a genomic imprinting model, which offers parental-specific gene expression. Using 3D FISH in porcine fetal liver cells, we compared the nuclear organization of the two parental alleles (expressed or not) of insulin-like growth factor 2 (IGF2), a paternally imprinted gene located on chromosome 2. We investigated whether its nuclear positioning favors specific locus associations. We also tested whether IGF2 is implicated in long-range chromatin *trans*-associations as previously shown in the mouse model species for its reciprocal imprinted gene H19.

**Results:**

We focused on the 3D position of IGF2 alleles, with respect to their individual chromosome 2 territories. The paternally expressed allele was tagged with nascent RNA. There were no significant differences in the position of the two alleles (*p* = 0.06). To determine long-range chromatin *trans*-interactions, we chose 12 genes, some of which are known to be imprinted in mammalian model species and belong to a network of imprinted genes (i.e. SLC38A4, DLK1, MEG3, and ZAC1). We screened them and ABCG2, OSBP2, OSBPL1, RPL32, NF1, ZAR1, SEP15, GPC3 for associations with IGF2 in liver cells. All imprinted genes tested showed an association with IGF2. The DLK1/MEG3 locus showed the highest rate of colocalization. This gene association was confirmed by 3D FISH (in 20 % of the nuclei analyzed), revealing also the close proximity of chromosomes 2 and 7 (in 60 % of nuclei). Furthermore, our observations showed that the expressed paternal IGF2 allele is involved in this association. This IGF2-(DLK1/MEG3) association also occurred in a high percentage of fetal muscle cells (36 % of nuclei). Finally, we showed that nascent IGF2, DLK1 and MEG3 RNAs can associate in pairs or in a three-way combination.

**Conclusion:**

Our results show that *trans*-associations occur between three imprinted genes IGF2, DLK1 and MEG3 both in fetal liver and muscle cells. All three expressed alleles associated in muscle cells. Our findings suggest that the 3D nuclear organization is linked to the transcriptional state of these genes.

**Electronic supplementary material:**

The online version of this article (doi:10.1186/s12860-016-0113-9) contains supplementary material, which is available to authorized users.

## Background

Over the past 10 years, extensive studies on the functional organization of the nuclear space in mammalian cells have shown that the architecture of the interphase nucleus is clearly non-random and compartmentalized. Furthermore, this high-level organization involving chromosomes, subnuclear compartments or the nuclear envelope appears to be important in regulating gene expression [1–3]. One feature of this landscape involves the dynamic, transient interactions that occur between different nuclear components and that regulate gene expression and silencing. Most genes in eukaryotes are transcribed by RNA polymerase II (Pol II), and several studies have suggested that it participates in transcription factories that regulate transcription at particularly high rates [4–6]. Several studies have reported that transcriptionally active genes, often found at the periphery of their chromosome territories (CT), can interact in *cis* or in *trans* in these Pol II-enriched compartments via chromatin loops [7–9]. These loops enable distal genes to engage in chromosomal contacts, which are strongly correlated with their transcriptional activity. Some of these interactions may allow contacts between “enhancers” and “promoters” [10], and may also facilitate the transcription of coregulated genes in multigene complexes [8].

Progress in imaging and molecular techniques help detect these interactions; these technical advances include fluorescence *in situ* hybridization (RNA and DNA FISH) and molecular approaches such as the chromatin proximity-ligation assay called chromosome conformation capture (3C) [11] and its derivatives, including Hi-C, that scan the whole genome for sequences found close together in nuclei [12, 13]. Another genome-wide assay, the chromatin interaction analysis with paired-end-tag sequencing (ChIA-PET), can detect long-range chromatin interactions associated with Pol II [14]. However, the in-depth analyses of these global interactome datasets generated from a given cell population have revealed large heterogeneity due to cell-to-cell variability, pinpointing the need to combine these approaches with analyses at the cellular level [15].

In the case of imprinted genes, often found in clusters [16], long-range intra- and interchromosomal interactions have been found and have been linked to the parental origin of regulatory sequences, supporting the existence of long-distance regulatory mechanisms [17]. One conserved imprinted region containing insulin-like growth factor 2 (IGF2), an important gene coding for one of the major growth factors [18], and H19 has been particularly studied. These genes, located approximatively 100 kb apart on mouse chromosome 7, are separated by an imprinting control region (ICR). This ICR regulates IGF2/H19 expression such that IGF2 is transcribed only from the methylated paternal chromosome and H19 only from the unmethylated maternal chromosome [19]. These differentially methylated regions interact in *cis* and these interactions partition maternal and paternal chromatin into distinct loops leading to different specific patterns of 3D organization for each allele [20]. Several studies on different cell types have also revealed *trans*-interactions between the H19 locus and other genes located on different chromosomes [17, 21, 22], with imprinted genes being overrepresented in these interchromosomal interactions. However, the epigenetic status of the H19 ICR seems to determine the patterns of most interchromosomal interactions. Thus in neonatal liver, various approaches (4C, 3C data and FISH) on specific mouse crosses have confirmed that the absence of CCCTC-binding factor (CTCF) (due to the maternal ICR deletion) abrogates the interchromosomal association between IGF2/H19 and other regions on mouse chromosomes 6 and 18 [17]. Similarly, DNA interactions between H19 ICR and delta-like homolog 1 (DLK1) preferentially involve the expressed maternal H19 allele whereas the paternal allele expressing IGF2 participates in only a low percentage of nuclei [22]. IGF2 involved in myogenesis, has a major effect on muscle mass in livestock, underlining its importance for pig breeding research. A paternally expressed quantitative trait locus (QTL) affecting muscle growth, fat deposition and heart size has been mapped to the IGF2 region on porcine chromosome 2 (SSC2; *Sus scrofa domestica* chomosome 2) [23–25]. Given the important role of IGF2, we focused our interest on this paternally expressed locus to test whether it is also involved in *trans*-associations in fetal pig liver and muscle cells. We first verified the imprinted status of IGF2/H19 in porcine fetal cells using RNA FISH. IGF2 belongs to an imprinted gene network (IGN) of coregulated genes predominantly expressed in somatic stem cells [26]; we therefore selected five genes from this network: SLC38A4, DLK1, MEG3, RPL32, ZAC1 and GPC3 located on chromosome X to screen for potential patterns of interactions with IGF2. We also analyzed NF1, OSBP2, OSBPL1 and ABCG2 because 3C/4C assays and 3D FISH analyses show that they interact with the IGF2/H19 domain in mouse neonatal liver cells [17] and finally SEP15, ZAR1 as controls. Preliminary screening done using DNA FISH on porcine fetal liver cells showed that the two neighboring reciprocally imprinted genes, DLK1 and maternally expressed gene 3 (MEG3) had the highest rate of colocalization with IGF2. To further analyze this colocalization, we used 3D FISH experiments, combined with RNA-DNA FISH experiments and confocal microscopy analyses, to determine which alleles are involved in this association.

In addition, because the monoallelic expression of IGF2 provides an interesting model system to probe the relationship between gene expression and nuclear position, we analyzed the position of the two alleles (expressed or not) relative to their CTs to shed light on the relationship between nuclear architecture and transcription control.

## Methods

### Preparation of complex DNA Probes

#### Probes for DNA FISH

Bacterial artificial clones (BACs) containing genes were isolated from a porcine BAC library (Biological Resources Center-GADIE, INRA, Jouy-en-Josas, France: http://crb-gadie.inra.fr/) using specific primers designed with Primer3 software (http://primer3.sourceforge.net/) (Additional file 1: Table S1). Approximately 50 ng of BAC DNA was random-priming labeled by incorporation of dUTP Alexa Fluor using the Bioprime DNA labeling kit (Invitrogen, Cergy-Pontoise, France). For multiple-label experiments, we labeled DNA FISH probes directly with Alexa Fluor 488 or 568, or, if necessary, with Biotin-16-dUTP detected by immuno-FISH. Porcine chromosome paint probes (for chromosomes 2 (SSC2) and 7 (SSC7)) from flow-sorted chromosomes [27] were individually directly labeled by random priming with Alexa Fluor 568 (Invitrogen).

#### Probes for RNA FISH

IGF2 genomic DNA probe encompassing IGF2 exons 5 to 9 (~5 kb) was generated by PCR to detect nascent RNA. H19 RNA FISH was performed using two genomic DNA fragments encompassing H19 exon 1 and exons 2 to 5 (total ~ 2 kb). For DLK1 RNA detection, a pool of several PCR fragments from genomic DNA amplification including exons 2, 3, 4 and 5 (~13 kb) was used. Similarly, two genomic DNA fragments encompassing MEG3 exons 1, 2 and 3 (~5 kb) were used to detect MEG3 RNA. Labeling was performed by random priming with incorporation of dUTP Alexa Fluor for single RNA FISH or with biotin revealed after hybridization by using the tyramide signal amplification kit (TSA^TM^ kit #22 with HRP–streptavidin and Alexa Fluor® 488, Invitrogen) for combined RNA/DNA FISH experiments.

Products from the labeling reactions (except the painting probes) were subsequently filtered through Microspin G-50 columns and ethanol precipitated with porcine Cot-1 DNA (Applied Genetics Laboratories, Melbourne, state, USA) and salmon sperm DNA (Eurobio, Les Ulis, France). Each probe was deposited on slides at a final concentration of 90 ng/μl in hybridization buffer (50 % formamide, 10 % dextran sulfate, 2× SSC).

### Preparation of cells and slides

Liver cells were prepared from fetal European Large White pig (90 days). All reagents were RNase-free and all steps performed at 4 °C to preserve RNA. After a brief fixation of 30 min in 4 % paraformaldehyde according to the size of the excised tissue (1 to 2 cm^3^), rinsed fresh liver tissues were partially disrupted to make a cell suspension in ice-cold phosphate-buffered saline (PBS) containing 5 mM vanadyl ribonucleoside complex (VRC). Cells resuspended in 1× DMEM medium containing 20 % glycerol were stored at −80 °C. Before *in situ* hybridization experiments, stored cells were thawed slowly then rinsed many times in cold 1× PBS before permeabilization for 3 min with 0.5 % Triton X-100 in cytoskeleton extraction buffer CSK (100 mM NaCl, 300 mM sucrose, 3 mM MgCl_2,_ 10 mM PIPES pH 6.8). After washes in 1× PBS, cells were postfixed for 2 min in cold 4 % paraformaldehyde. About 15 μl of a dense cell suspension obtained after centrifugation at 300 × g for 3 min were applied to Superfrost glass slides (CML, Nemours, France) to obtain cell adhesion just before hybridization experiments. To preserve 3D cell structures, preparations were not dehydrated with an ethanol series, but were just deposited on the slide and left to air-dry for a few minutes.

For the preparation of muscle samples, tissues were dissected to obtain a fiber pack and successive treatments as described above were carried out in small petri dishes.

### *In situ* hybridization experiments

#### 2D DNA FISH

The chromosomal localizations and specificity of all probes used (BAC-containing genes and chromosome paints) were controlled by 2D DNA FISH on porcine metaphases prepared from lymphocytes according to standard protocols [28]. IGF2 is localized on SSC2p17 and (DLK1-MEG3) on SSC7q26 (Additional file 1: Table S1).

#### 3D DNA-FISH experiments on interphase nuclei

*In situ* hybridization experiments were carried out immediately after cell preparation on slides. After probe solution was applied to the cell preparation and the coverslip sealed with rubber cement, cells and probes were simultaneously heat-denatured at 75 °C for 8 min and then incubated overnight at 37 °C in a wet chamber in a DAKO hybridizer. After removing the coverslips, post-hybridization washes were performed with gentle agitation first in 2× SSC at room temperature (three times) for 3 min, then twice for 3 min in 2× SSC, 50 % formamide pH 7.0 at 40 °C and finally, twice for 15 min in 2× SSC then in PBS at room temperature. When a biotin-labeled probe was used, the slides were blocked with 0.5 % PBS/BSA at room temperature before biotins were detected by incubating the slides with streptavidin-Alexa 633 for 1 h at room temperature. The slides were washed in PBS/Tween-20 (0.1 %) for 2 × 10 min and PBS 3 × 15 min at 37 °C. Nuclei were counterstained with 4′,6′diamidino-2- phenylindole (DAPI) in Vectashield medium (Vector Laboratories, Burlingame, CA, USA).

#### 3D RNA-DNA FISH

RNA and DNA FISH were performed sequentially on the same nuclei and visualized in 3 or 4 different colors. Probes for RNA FISH were denatured for 6 min at 90 °C and immediately put on ice. After 10 min, RNAsin was added to the probes before depositing on slides. Non-denatured cells were then hybridized overnight in a humidified environment at 37 °C in a hybridizer. Post-hybridization washes were performed in 2 × SSC at room temperature (four times) for 15 min. To increase the sensitivity and enhance the stability of the RNA signal during the subsequent DNA FISH, a tyramide-Alexa 488 signal amplification kit (TSA) was used. Briefly hybridized biotinylated DNA probes are saturated with streptavidin-horseradish peroxidase (HRP). In the presence of small amounts of hydrogen peroxide, streptavidin-HRP converts a labeled tyramide into an extremely reactive intermediate resulting in minimal diffusion-related loss of signal localization. The TSA kit was used following the protocol provided by the manufacturer. Before hybridization, some slides were treated with 200 μg/ml RNase for 1 h at 37 °C as negative controls. Just before the subsequent DNA FISH experiment (following the same protocol as described above), cells were slightly permeabilized again with 0.25 % Triton X-100 in CSK for 3 min maximum to favor access of Alexa 633-streptavidin to the nuclear space and were then washed in PBS (twice, 10 min each wash).

When four-color RNA-DNA FISH was performed, we labeled the probes as follows: the RNA signals were detected using biotinylated probes revealed by Alexa 488-TSA kit, porcine chromosome painting probes were preferentially directly labeled by random priming with Alexa 568 (Invitrogen) and other DNA probes by indirect labeling with biotin-streptavidin conjugated with Alexa 633. Sequential RNA and DNA FISH allowed us to use two biotinylated probes without detection problem. During the first step (RNA FISH), the TSA system totally saturates the biotinylated probe and stabilizes the signals during the revelation process, thereby allowing immediate reuse of the cells for DNA FISH with another biotinylated probe.

### First screening of DNA interactions

The 3D DNA FISH protocol was used but the analysis was done on a Zeiss Axiophot epifluorescence microscope coupled to a Cytovision workstation (Leica Biosystems). Loci were considered as associated when signals were observed in close proximity (touching each other) and/or colocalized. No distance measurement was obtained in this case.

### Confocal microscopy and image analyses

Image stacks were collected using a Leica TCSSP2 confocal microscope (Leica Instruments, Heidelberg, Germany) equipped with an oil immersion objective (plan achromatic 63× N.A. = 1.4). The Z-stacks (confocal planes) were acquired at 1024 × 1024 pixels per frame using an 8-bit pixel depth for each channel at a constant voxel size of 0.077 × 0.077 × 0.284 μm.

Segmentations and 3D measurements between objects (nucleus, genes, RNA, nucleolus and CT) were done using NEMO [29] as described previously [30], an ImageJ plug-in designed to interactively analyze FISH 3D images and automatically measure object distances in multiple-channel experiments. The program distributed under the creative commons license can be freely downloaded from https://forge-dga.jouy.inra.fr/projects/nemo. We carried out our analyses on about 60 confocal planes in semi-automatic detection mode to minimize image signal-to-noise ratio and keep only the informative signals inside the nucleus for further processing. After having determined the processing parameters that define xyz resolutions and filters to run on raw images (3D median and 3D mathematical morphology filters applied to all objects and TopHat filter applied to the gene channel), all objects within a nucleus were detected automatically based on the intensity of pixels above a globally set threshold. The criteria for segmented object validation (SNR minimum and maximum values, object volume (minimum and maximum) and number) were defined once for each type of object and applied to all nuclei. The resulting segmentation for each cell was validated by manual comparison with the raw image. NEMO can compute the percentage of colocalization and various distances between objects (spots, CTs, nucleoli or nuclei): center-to-center distances, center-to-border or border-to-border distances between objects. The distances computed were Euclidean distances taking into account the x, y and z resolutions. Given the resolution on the z axis, at least three pixels corresponding to 0.852 μm (0.284x3) were required for a high resolution between two separate signals; consequently 1 μm was chosen as the upper cut-off for associated signals.

### Spatial positioning of IGF2 RNA or DNA relative to chromosome territories

For each cell, we measured the distances between RNA or DNA spot centers and CT edge and the percentage of colocalization in the CTs. To determine the position of the signals (RNA or DNA) relative to their CT, we defined three categories (inside, edge, outside) as described previously [30], taking into account the allele center-to-CT edge distances and the percentage of gene colocalization in CT. The edge category comprises genes located at less than one voxel from the edge of the segmented CT. A *χ* ^2^ test was used to compare the signal distribution in these categories. *P* values < 0.05 were considered as significant.

### Gene-gene or RNA-gene associations

The 3D distances (center-to-center) between loci or loci-RNA were measured to determine if associations occur between them. A distance of 1 μm was chosen as upper cut-off for association. Signals were classified into two different categories: 1) associated, when the two loci were found separated by a distance d ≤ 1 μm that generally corresponded to partially colocalized loci; 2) distant, when d > 1 μm.

## Results

### Analysis of IGF2 RNA and DNA nuclear organization

#### IGF2 and H19 imprinting status in fetal liver cells

**Fig. 1:**
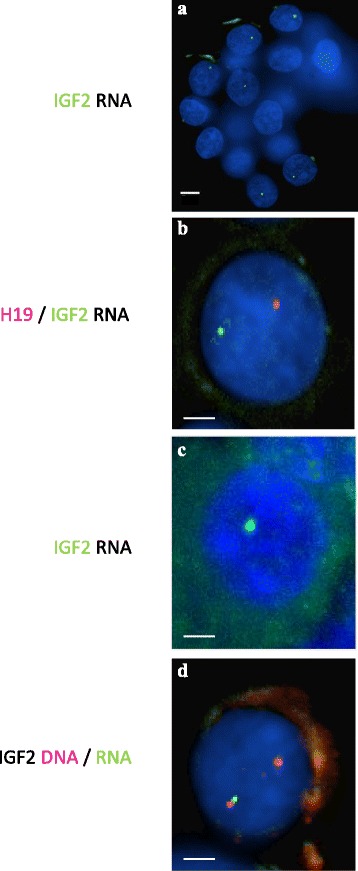
RNA and successive DNA FISH of imprinted IGF2 locus in porcine fetal liver cells. Nuclei are counterstained with 4′,6′ diamidino-2-phenylindole (DAPI, blue). **a**, IGF2 RNA FISH: monoallelic expression of IGF2 (one spot labeled in green per nucleus) is detected when non-denatured nuclei are hybridized with a direct Alexa488 labeled IGF2 probe. The monoallelic pattern was detected in six nuclei. Bar = 5 μm; **b**, IGF2 and H19 RNA FISH: monoallelic expression of IGF2 (one spot labeled in green with dUTP-Alexa488) and H19 (one spot labeled in red with dUTP-Alexa568). IGF2 and H19 RNAs were distant from each other, corresponding to the transcripts of single expressed allele from opposite chromosome 2; **c**, IGF2 RNA FISH: IGF2 RNA signal was enhanced and stabilized by the use of TSA kit (Biotin - TSA - Alexa488 label); **d**, Sequential IGF2 RNA-DNA FISH: IGF2 RNA was labeled with a Biotin-TSA-Alexa488 probe (one green spot) and IGF2 DNA with a direct Alexa568-labeled BAC probe (2 spots in red). The RNA signal tags the expressed allele. Bar = 2 μm

We set up RNA FISH experiments on non-denatured porcine fetal liver cells using an IGF2 probe to verify its imprinted status. We first verified that the signals detected corresponded to RNA spots (all signals were sensitive to RNase treatment and no signal was obtained when the probe was hybridized on denatured cells). The biallelic expression of β actin was used as a positive control (data not shown). IGF2 imprinting status was analyzed in 5 pigs (*n* > 140 for each animal; total number of nuclei analyzed, *n* = 795). IGF2 expression was detected in 64 % of the liver cells investigated, and observed as a single spot in the vast majority (97 %) of these labeled nuclei, thereby confirming its monoallelic expression status (Fig. 1a). The parental origin of the transcripts cannot be determined in RNA FISH experiments. However, exclusive paternal IGF2 expression in porcine fetal liver cells has been demonstrated previously [31]. We also verified the monoallelic expression of H19 (tightly linked to IGF2, but with reciprocal imprinting) in fetal liver cells. The majority of cells displayed a monoallelic transcription pattern (98 % on 77 nuclei). Both H19 and IGF2 RNAs can be detected in the same nucleus as illustrated in Fig. 1b.

#### IGF2 positioning relative to SSC2 chromosome territories

**Fig. 2:**
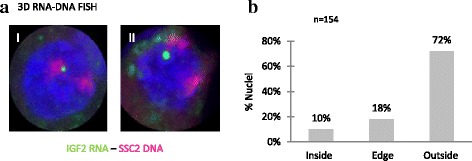
Analysis of nuclear positioning of IGF2 transcripts relative to the proximal chromosome territory (SSC2) in interphase nuclei of porcine liver cells by 3D FISH and confocal microscopy. **a**, Two images of 3D RNA-DNA FISH revealing IGF2 RNA signals (*green*) and SSC2 (*red*): (I: IGF2 RNA at the edge SSC2 and II: IGF2 RNA outside SSC2). Nuclei were counterstained with DAPI; **b**, The distribution of IGF2 RNA towards the chromosome territory (SSC2) was scored in one of three classes: inside, edge and outside. The three classes were defined by combining the 3D distances (RNA spot center to chromosome territory edge and the percentage of colocalization (RNA in its chromosome territory) obtained with NEMO [29]. A significant fraction of RNA signals was found located outside SSC2 (in 72 % of nuclei)

To determine the position of nascent RNA relative to the edge of SSC2 CT, transcription sites relative to the SSC2 CT were analyzed by using sequential RNA-DNA FISH. The RNA signals were amplified and stabilized using a TSA kit (Fig. 1c and d). According to the criteria presented in Methods, nascent IGF2 RNAs were preferentially located outside their CTs in 72 % of the 154 nuclei analyzed (Fig. 2a and b). The maximal distance found between the center of an RNA signal and the CT edge reached 3.5 μm.

**Fig. 3:**
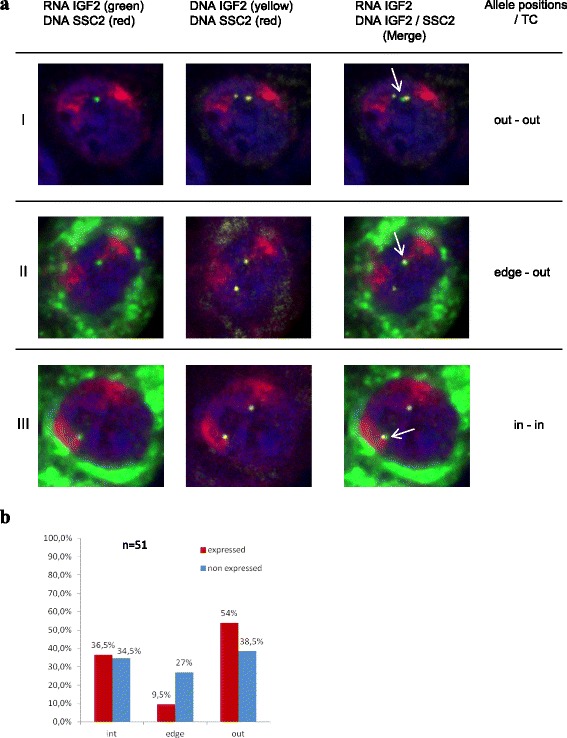
Position of expressed and non-expressed IGF2 alleles, relative to chromosome territories (SSC2). **a**, Extended focus of image sections from confocal microscopy created using Volocity software illustrating three scenarios (lines I, II, III) of IGF2 allele position towards SSC2: (I (out-out), II (edge-out) and III (in-in)). IGF2 gene transcription sites were detected by 3D RNA FISH, IGF2 DNA alleles and SSC2 by 3D DNA FISH. Column 1 shows the IGF2 RNA (*green*) and SSC2 (*red*), column 2 IGF2 DNA (*green*) and SSC2 (*red*), column 3 the merge. The nuclei were counterstained with DAPI (*blue*), the green fluorescence outside the nucleus is due to autofluorescence. White arrows on merge images indicate the position of the expressed alleles based on RNA FISH signals; **b**, Histogram representing the percentage of nuclei of the two groups of IGF2 alleles (expressed or not) relative to SSC2 (inside, edge or outside) in 51 nuclei

The monoallelic expression of IGF2 made it possible to probe the relationship between gene activity and nuclear positioning by comparing, in a single cell nucleus, the position of the active and inactive alleles relative to CTs. We developed a four-color 3D RNA-DNA FISH method to detect simultaneously the nascent IGF2 RNA molecules, the two IGF2 DNA loci, the CTs and the nucleus. Because this type of four-color 3D experiment is difficult to implement, we proceeded by steps. We first combined DNA and RNA FISH experiments to visualize the two DNA alleles simultaneously as well as the transcription sites labeling the active allele. We observed only one IGF2 RNA signal (nascent RNA) close to one of the two DNA loci (Fig. 1d). We then added the chromosome painting probe to label the chromosome 2 territories (Fig. 3a). We assigned each allele to its CT by identifying the nearest signal in 51 nuclei. The distance between the center of the DNA signal and the CT edge and the percentage of allele colocalization in the CT were determined for each allele. Three categories (inside, on the edge of and outside the CT) were defined to classify the allele position relative to the CT. Two groups (expressed and non-expressed alleles) were assessed. There was no specific preferential localization for the alleles according to their expression status (*χ*2 test, *p* = 0.06), although expressed alleles showed a slight tendency to be located outside their CT (Fig. 3b). The measurements of 3D (DNA spot center-CT edge) distances for both alleles ranged from 0 to 2.5 μm and confirmed the tendency of the expressed allele to be more frequently outside the CT (Additional file 2: Figure S1a). We then analyzed the data nucleus by nucleus to study pairs of alleles. However, the total number of nuclei (*n* = 51) was insufficient to obtain a robust conclusion considering the nine allele position combinations (Additional file 2: Figure S1b).

### Screening for genes that potentially associate with IGF2

In mammal model species, the clustering of imprinted genes is linked to common and long-distance regulatory mechanisms, including *trans*-interactions that contribute to the regulation of gene expression. Our aim was to determine if IGF2 associates with other genes in porcine fetal tissues. We selected genes, some imprinted (denoted i) and/or also highly expressed in fetal liver cells (denoted e): SLC38A4 (i,e), DLK1 (i,e), MEG3 (i,e), RPL32 (e), ZAC1 (i,e), GPC3 (e), NF1, OSBP2, OSBPL1 and ABCG2. The latter four genes interact with the IGF2/H19 domain in mouse neonatal liver cells [17], but information on their expression level in porcine fetal liver is known only for OSBPL1 and ABCG2 (weakly expressed, L. Liaubet personal communication). We carried out a preliminary screening as described in Methods. The results of association between IGF2 and these genes are given in Table 1.

**Table 1:** Detected gene associations upon preliminary screening on liver cells

IGF2 in association with	Number of nuclei analyzed	Percentage of nuclei with		
		Distant signals	Associated (close + colocalized) signals	
(DLK1-MEG3)^a^	125	65 %	35 %	(21 % + 14 %)
NF1	106	63 %	37 %	(24 % +13 %)
OSBP2	108	71 %	29 %	(17 % + 12 %)
OSBPL1	79	81 %	19 %	(8 % + 11 %)
ABCG2	124	71 %	29 %	(19 % + 10 %)
SLC38A4^a^	219	76 %	24 %	(15 % + 9 %)
ZAC1^a^	89	80 %	20 %	(5 % + 15 %)
RPL32	109	93 %	7 %	(2 % + 5 %)
GPC3	82	91 %	9 %	(5 % + 4 %)

^a^ Genes imprinted in pig

The percentages of association ranged from 7 to 37 %, and were relatively high (≥19 %) for seven of the nine investigated genes, suggesting that IGF2 associates with some of these genes. It includes the four genes previously described to interact with the IGF2/H19 domain in mouse neonatal liver cells. The highest values of association frequency (around 35 %) were observed between IGF2 and (DLK1-MEG3) and between IGF2 and NF1. Of these associations, we focused on the association between the imprinted IGF2 and DLK1-MEG3 (DLK1 paternally and MEG3 maternally expressed) genes to validate and further investigate it using a 3D analysis.

### 3D analysis of *trans*-association between IGF2 and (DLK1-MEG3)

#### Determination of non-associating control

Relying on the fact that transcribed genes tend to interact preferentially, we selected two genes ZAR1 (mapped to SSC8) and SEP15 (mapped to SSC4) expressed at a very low level in liver and muscle cells (L. Liaubet, personal communication) as negative controls. We first verified that these genes were associated only in a low percentage of cells. ZAR1 associated with SEP15 in only 7 % of the cells analyzed (*n* = 61), and never at a distance <1 μm. Second, we tested the non-association between IGF2 (highly expressed) and ZAR1 (weakly expressed). IGF2 was found associated with ZAR1 in 8 % of the cells analyzed (*n* = 56).

#### The expressed IGF2 allele associates in *trans* with (DLK1-MEG3) in liver cells

**Fig. 4:**
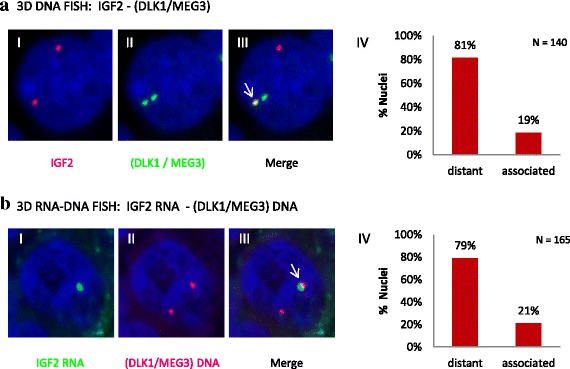
Analysis of the association between IGF2 and (DLK1-MEG3) in liver cells. Decomposed confocal images (maximal intensity projections) were processed using Volocity software. The nucleus was counterstained with DAPI (*blue*). **a**, 3D DNA FISH: IGF2 – (DLK1/MEG3). I: IGF2 alleles (*red*), II: (DLK1-MEG3) alleles (*green*), each green spot corresponds to both merged alleles of DLK1 and MEG3 genes, III: merge, white arrows point to associated signals. On the right (IV)**; b,** 3D RNA-DNA FISH: IGF2 RNA – (DLK1/MEG3) DNA. I: IGF2 RNA (*green*), II: (DLK1-MEG3) alleles (*red*), III: merge, white arrows point associated signals. On the left (IV): Histogram presenting the percentage of nuclei with RNA-DNA association was shown

We first confirmed the association between IGF2 and (DLK1-MEG3) loci by performing 3D DNA FISH (Fig. 4a). DLK1 and MEG3, located in the same BAC clone due to their close proximity, were visualized as one signal. An association between IGF2 and (DLK1-MEG3) regions was detected in 19 % of the nuclei investigated (*n* = 140). Only one IGF2 allele was associated with one (DLK1-MEG3) allele. Given that the DLK1-MEG3 and IGF2 loci map to different porcine chromosomes (i.e. SSC7q26 and SSC2p17, respectively), this interchromosomal association suggests a spatial proximity of SSC2 and SSC7. These CTs were found in close proximity (edge to edge) in 60 % of the nuclei analyzed (*n* = 80). Among them, each SSC2 chromosome was found close to a SSC7 in 17.5 % of the nuclei and only one SSC2 was found close to a SSC7 in 42.5 % of the nuclei. An illustration of the co-hybridization of chromosome paints (SSC2 and SSC7) and gene clusters (IGF2 and DLK1/MEG3) in a multi-color 3D DNA FISH experiment is shown in Additional file 3: Figure S2.

To determine if one particular IGF2 allele is involved, we tagged the expressed allele by labeling the nascent IGF2 RNA in a three-color 3D RNA-DNA FISH experiment allowing the simultaneous visualization of IGF2 RNA signals and DLK1 DNA spots (Fig. 4b). Signals were found associated in 34 out of 165 nuclei analyzed corresponding to 21 % of nuclei. The results of the frequencies of *trans*-associations in pig fetal cells are reported in Table 2. Percentages of cells showing association between IGF2 and DLK1, both in DNA-DNA and RNA-DNA experiments were concordant (19 and 21 %). The last experiment highlighted that this association involved the expressed IGF2 allele but, due to the low expression level of DLK1 in fetal liver cells, it was not possible to determine by RNA FISH if the association involves the expressed allele of DLK1, of MEG3 or both. As IGF2/H19, DLK1 and MEG3 reciprocal imprinted genes have been shown to be highly expressed in porcine fetal muscle ([32], L. Liaubet personal communication), we completed the analysis in this tissue.

**Table 2:** Percentage of cells showing *trans*-associations in pig fetal liver and muscle cells

Fetal tissue	3D-FISH Experiment	*trans*-associations	Number of nuclei analyzed	Percentage of nuclei with associated signals (d ≤ 1 μm)
Liver	DNA	IGF2 + (DLK1/MEG3)	140	19 %
Liver	RNA-DNA	IGF2 + (DLK1/MEG3)	165	21 %
Muscle	DNA	IGF2 + (DLK1/MEG3)	60	36 %
Muscle	RNA	IGF2 + DLK1	57	32 %
Muscle	RNA	IGF2 + MEG3	80	40 %
Muscle	RNA	MEG3 + DLK1	63	25 %
Muscle	RNA	MEG3 + IGF2 + DLK1	24	13 %
Muscle	DNA	IGF2 + ZAR1	61	8 %
Muscle	DNA	IGF2 + SEP15	56	7 %

#### Deciphering the association between the expressed IGF2 allele and the DLK1/MEG3 alleles in muscle cells

**Fig. 5:**
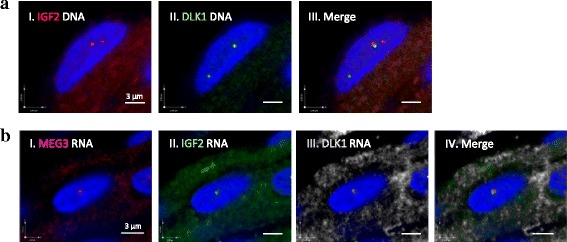
Analysis of the association between IGF2 and (DLK1-MEG3) in muscle cells. Decomposed confocal images and merge images are shown. The nucleus is counterstained with DAPI (*blue*). Bar = 3 μm. **a**, 3D DNA FISH showing DNA hybridization signals. I: IGF2 DNA (*red*), II: (DLK1-MEG3) (*green*) DNA and III: merge. Only one allele of IGF2 was associated with one allele of (DLK1-MEG3) region; **b**, 3D RNA FISH. I: MEG3 RNA (*red*), II: IGF2 RNA (*green*) and III: DLK1 RNA (*white*). The three IGF2 (*green*), DLK1 (*white*), MEG3 (red) RNAs were shown partially colocalized

We first verified that the DNA-DNA association found between IGF2 and DLK1-MEG3 loci in liver cells was also conserved in fetal muscle cells. This association was detected in 36 % of the nuclei investigated (*n* = 60). In the great majority of cells in which this association was observed (77 %), it involved only one allele of each locus (Fig. 5a). Other patterns (two alleles of one gene associated with two alleles of the other, one allele of one gene associated with the two alleles of the other) were underrepresented (< 3 %). In addition, the two alleles of the (DLK1-MEG3) locus were associated in 10 % of nuclei.

We then carried out DLK1 and MEG3 RNA FISH experiments in muscle. The transcription of these genes was observed in approximately 50 % of the cells analyzed (*n* = 100). By using RNA combined with DNA FISH, we confirmed the monoallelic expression status of IGF2, DLK1 and MEG3 in 95, 90 and 89 % of muscle cell nuclei respectively, all detected close to their DNA loci. To determine if the expressed alleles of these three different imprinted genes associate, we performed 3D RNA FISH experiments. IGF2 and DLK1 RNA signals were associated in 32 % of the nuclei analyzed (n = 57), IGF2 and MEG3 RNAs in 40 %, including a high percentage of colocalized signals (25 %) (*n* = 80 nuclei) demonstrating that the expressed IGF2 allele associated with both expressed DLK1 and MEG3 alleles (Table 2). MEG3 and DLK1 RNA transcripts were also associated in 25 % of the nuclei analyzed (*n* = 63), confirming the association observed in the DNA FISH experiment. A multiple labeling experiment was carried out to analyze the three RNAs simultaneously. The three RNA signals were found associated in 12.5 % of the nuclei (*n* = 24) (Fig. 5b). A double RNA association was detected in 40 % of the nuclei for IGF2/MEG3 (*n* = 80), 25 % of the nuclei for DLK1/MEG3 (*n* = 63) and 32 % of the nuclei for IGF2/DLK1 (*n* = 57) (Table 2, Additional file 4: Figure S3).

#### Migration of H19 nuclear RNA in muscle cells

**Fig. 6:**
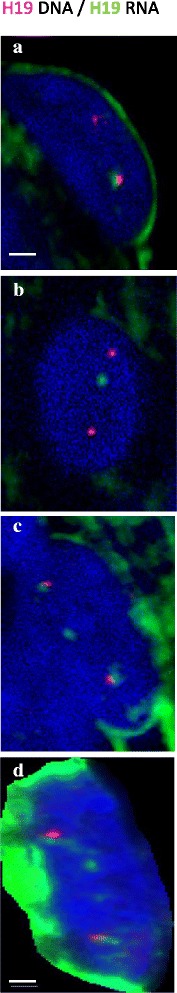
Analysis of H19 hybridization patterns (DNA and RNA) in muscle cells. 3D RNA-DNA FISH: H19 RNA spot was labeled in green and H19 DNA loci in red, nucleus is counterstained with DAPI. Bar = 1 μm. **a,** H19 RNA spot near one H19 DNA locus; **b**, one H19 RNA spot distant to the two DNA loci; **c,** two H19 RNA spots located at DNA loci and an additional migrating H19 RNA signal; **d**, H19 RNA signals as a beads-on-string pattern migrating from one H19 allele to the other

Our aim was to label the maternal IGF2 allele to determine if the non-expressed allele is also implicated in associations with other loci. One possible way to label the non-expressed IGF2 allele is to target the reciprocally imprinted H19 allele. We carried out a 3D RNA-DNA FISH experiment with different probes labeling H19 RNA and H19 DNA loci. The analysis was more complex than expected (Fig. 6). Although 83 % of the nuclei analyzed (*n* = 64) showed a single H19 RNA spot (Fig. 6a), this spot was found in proximity to the corresponding DNA locus (site of RNA synthesis) in only 22 % of the cells. In most of the cases (61 %), it was located far away, suggesting that it had migrated in the nuclear space (with a distance greater than 2 μm in 19 % of analyzed nuclei) (Fig. 6b). In addition, at least two spots were detected in the remaining 17 % of the nuclei analyzed. In this case, two patterns were observed: 1) two RNA spots (nascent RNA) were detected close to the two DNA loci (6 % of nuclei); 2) more than two spots were observed including at least one spot distant from the DNA locus (11 % of nuclei) (Fig. 6c). Remarkably, 72 % of all the nuclei analyzed showed an RNA spot distant from the DNA locus, suggesting a migration phenomenon. We also observed a beads-on-a-string pattern of H19 RNA signals between the two H19 alleles (Fig. 6d). Consequently, we were not able to use H19 RNA signals to label the IGF2 maternal allele.

## Discussion

### Detection and position of IGF2 and H19 RNAs in porcine fetal tissues

The imprinting of the tandem IGF2-H19 locus is required for their balanced expression and normal development [33]. Paternally expressed IGF2 acts as a growth factor and maternally expressed H19 non-coding RNA regulates the transcription of growth-enhancing (including IGF2, DLK1 and MEG3) and growth-inhibiting imprinted genes during gestation. Regulation of genomic imprinting at this locus is well established and is expected to be conserved among species. In pig, the IGF2-H19 imprinting cluster is quite similar to the corresponding well-studied human cluster [31, 34, 35]. It also exhibits several striking features, including a very high GC content (greater than 55 %) and an exceptional concentration of CpG islands often associated with ICR. Paternal imprinting status of IGF2 has previously been studied in porcine liver and muscle [25, 31] and H19 has been shown to be exclusively expressed from the maternal allele in all major organs [36]. The paternal expression of DLK1 and the maternal expression of MEG3 in muscle tissue have been also confirmed in pig [37]. Our RNA FISH results are in agreement with these data: the majority of fetal liver and muscle cells displayed monoallelic expression for IGF2, DLK1 and MEG3 detected at their respective DNA locus positions, thereby discriminating between the two parental alleles. H19 was clearly monoallelically expressed in fetal porcine liver cells. However, its imprinting status in fetal muscle cells was difficult to determine because we detected more than one RNA spot in a high percentage of cells (about 80 %): these spots were either located close to DNA alleles and/or elsewhere in the nucleus. There are two plausible explanations: 1) the expression of H19 in this tissue and at this stage of development is not strictly monoallelic; 2) H19 RNA signals may migrate in a condensed form into smaller foci, adopting a beads-on-a-string pattern within the nucleus. The maternal H19 allele expresses several RNAs including a 2.3 kb long non-coding H19 RNA (H19 lncRNA) and a large antisens H19 nuclear transcript (91H RNA). In mouse, lncRNA has been shown to be an effective player that functions as a trans-regulator that can silence nine target coexpressed genes of the IGN (including IGF2), likely important for fetal development and early postnatal growth control [38, 39]. The 91H RNA is another player in IGF2 transcriptional regulation, but it is a short-lived nuclear transcript found in small quantities relative to H19 lncRNA. The most likely hypothesis is that we visualized migration of H19 lncRNA towards their targets. Therefore, we were not able to use the H19 RNA spots to label the maternal allele.

### IGF2/H19 nuclear organization

One of many mechanisms that control gene expression, dynamic gene repositioning in the nuclear space in response to different cell stimuli optimizes the regulation of gene expression. In particular, transcriptionally active genes are positioned on the edge of a CT and tend to relocate to specialized subnuclear compartments [40–43]. Thus, correlations between gene repositioning and transcriptional status have been well demonstrated for specific genes that switch from the silent to the active state during differentiation and development processes [44–46]. Monoallelically expressed genes are useful for investigating the relationship between nuclear positioning and gene activity by the comparison of the nuclear position of active and inactive alleles within the same cell nucleus. Various examples have provided some evidence for function-related differential positioning of alleles within the interphase nucleus [47, 48]. For example, Takizawa et al. [49] observed a significantly different radial position (more internally positioned) of the stochastic expressed IL4 and adipocyte marker GFAP alleles compared with their respective non-expressed alleles. Similarly, Gribnau et al. [50] found cell-type-specific differences in the nuclear localization of the two expressed parental IGF2 and H19 alleles in mouse embryonic stem (ES) and fetal liver cells. In this study, we focused on the localization of alleles with regard to their own CT. We identified the position of the expressed IGF2 alleles, with a tendency to be outside the SSC2 CT. However, there was no significant statistical difference between the two alleles probably because IGF2, like many other imprinted genes, is embedded in a gene-dense chromosomal region with about 10 adjacent imprinted genes, including maternally expressed imprinted genes. Therefore, it is important to consider the whole environment of the genomic locus rather than only the gene itself to fully understand its 3D architecture.

### *trans*-association with IGF2

We observed that IGF2 was frequently positioned at the edge of the CT. This position may favor *trans*-associations with other loci important for gene regulation. In this study, we focused on *trans*-associations involving IGF2 and found three imprinted genes located on different chromosomes (IGF2 on SSC2) and (DLK1 and MEG3 on SSC7) physically associated or colocalized in the nucleus. The expected frequency of random colocalization has been defined to be less than 1 % [41, 51]. Sandhu et al. determined an average interaction frequency of 2 % for controls [22]. In our experiment, non-expressed genes chosen as negative controls were found associated in 7 % of cells (the lowest percentage that we obtained). However, others [17] consider that *trans*-interactions detected in 8 % of the cells are significant. This raises the question of the difficulty in choosing non-associating controls. Nevertheless, the high frequency of the associations found in this study clearly indicate their significance.

In the past 10 years, gene interactions have been studied on the whole genome scale by derived 3C approaches [12, 52]. Interestingly, imprinted genes have been shown to be overrepresented among the regions implicated in intra- and inter-chromosomal interactions despite their small number (~100 genes). This overrepresentation and the non-probabilistic nature of allelic expression suggest that epigenetic mechanisms — such as the different methylation status of the ICR in paternal and maternal alleles, their different CTCF binding sites and allele-specific patterns of 3D organization [20, 53] — are probably involved in their propensity to associate with other loci. Thus, several studies on different mouse cell types, have revealed *trans*-interactions between IGF2/H19 and other genes, suggesting that physical networks of imprinted genes are relatively common [17, 21, 22]. Our data are in agreement with this hypothesis because all tested imprinted genes (DLK1, MEG3, SLC38A4 and ZAC1) showed a high percentage of association with IGF2 and these genes were also highly expressed in pig. This result suggests that imprinting and high expression drive these associations. In neonatal mouse liver, Zhao et al. [17] found *trans*-interactions between the IGF2/H19 domain (chromosome 11) and ABCG2 (chromosome 6) and OSBPL1A (chromosome 18) in 8 to 12 % of cells, respectively. In mouse ES cells and neonatal liver, seven imprinted genes have been shown to interact in a pairwise manner in 3 to 14 % of the nuclei [22]. By using the same criteria of 3D center-to-center distances, we showed that IGF2 and DLK1/MEG3 loci associated in 19 to 36 % of the fetal porcine liver and muscle cells respectively.

By performing 3D RNA/DNA FISH, we showed that it is the paternal IGF2 allele that associates frequently with the DLK1/MEG3 locus in fetal liver (21 %) and muscle cells (36 %). Although available previous data have shown that the maternal H19 ICR is preferentially involved in *trans*-interactions [17, 21, 22], and particularly with DLK1 [22], our results demonstrate that the paternally expressed IGF2 allele is also clearly involved in *trans-*interactions. The higher percentage obtained in fetal tissue may be related to the fact that the levels of expression of these genes are higher at fetal stages than in neonatal stages in pigs and sheep [25, 54]. We also detected pairwise RNA associations: (IGF2/DLK1), (MEG3/IGF2) in relatively similar percentages of cells (32 %, 40 %) respectively and a triple association (IGF2/DLK1/MEG3) in 13 % of cells. This result illustrates that the paternal IGF2 allele associates with both DLK1 and MEG3 loci located on the two homologous SSC7. Simultaneous interactions between more than two imprinted domains called a “party interaction” between H19, INS1, DLK1 DNAs have been reported; their low frequency (6 % of cells) is attributed to the dynamic, transient character of the association [22]. It would be interesting to verify if these contacts depend on each other and occur in common specialized transcription factories [55, 56], as demonstrated previously in erythroid cells [57]. Within the NFkB-regulated multigene complex, perturbation disrupting a loop-mediated contact may drastically affect the transcription of other interacting genes involved in both *cis* and *trans* contacts [8]. Thus, there is evidence that such chromatin looping is regulated in a hierarchical manner, suggesting that gene looping has a significant impact on the cotranscription of some interacting genes. This cooperation between active coregulated genes may boost the expression of these genes.

In mammalian cells, interaction studies based on Hi-C approaches nevertheless reveal that genomic proximity is not the only factor. Distinct chromosomal domains tend to have their own preferred interaction partners: active ones preferentially interacting with other active ones (GC-rich domains associate preferentially with other GC-rich regions) through a partitioning of chromosomes into topologically associated domains (TADs) [46, 58]. Our data are in agreement with these observations: IGF2 and DLK1 loci are located in gene-dense chromosomal regions called regions of increased gene expression (RIDGEs) [59].

Our data demonstrate that SSC2 and SSC7 were close together in a high percentage of liver cells compared with associating IGF2-(DLK1/MEG3) loci, suggesting that chromatin looping may exist before target gene expression (and thus influences it) and/or persist after transcription as previously suggested [60, 61]. We hypothesize that the proximity of CT promotes locus interactions. However, to confirm this hypothesis some additional information is needed: the estimation of the probability that two loci interact randomly based on the proximity of their CT and the analysis of other loci on these CT.

Otherwise, the degrees of intermingling between CTs, the fragility of the decondensed chromatin loop in transcription-active domains, have been shown to be functionally relevant in determining the outcome of translocation [62]. Several cytogenetic studies, especially in cancer research, have shown that translocation-prone gene partners are preferentially found in close spatial proximity in normal cells before translocation events [63–65]. *Trans*-interactions and chromosomal translocations seem to be correlated, especially when transcriptional loci present some sequence homology [66–68]. In our case, the IGF2/H19 imprinted cluster is shown to share many features (DMRs, CTCF binding sites) with the DLK1/MEG3 region. Through the chromosomal diagnostic activities in our laboratory, we have found two translocations involving the identified chromosomal regions t(2p;7q) carrying respectively IGF2 and DLK1 (A. Pinton, personal communication). Knowledge on 3D nuclear organization, especially of active genes that tend to cluster, will help shed light on chromosome translocation mechanisms by identifying gene partners [69].

## Conclusion

Our results provide a view of the spatial organization of IGF2 alleles in porcine fetal liver and muscle cells. In regard to their CTs, no differences in 3D positions was found between expressed and non-expressed IGF2 alleles in liver cells. Focusing on the expressed IGF2 allele using 3D RNA-DNA FISH, we report three significant findings. Firstly, we confirmed the widespread observations of physical associations between imprinted domains in different tissues (fetal liver and muscle) and species including pig. Secondly, we completed previous studies that showed a preferential maternal H19 ICR implication in interactions by demonstrating that the paternal IGF2 allele is also highly involved in *trans*-associations with the DLK1/MEG3 region. Together, our results suggest that the two parental IGF2/H19 regions are involved in gene associations. Thirdly, we investigated these associations and showed that the paternally expressed IGF2 allele associates simultaneously with both the paternally expressed DLK1 and maternally expressed MEG3 alleles in muscle cells.

Although most studies on imprinting have been performed on mouse models or in the context of human biomedical disorders, imprinted genes hold promise for research in domestic livestock for their putative major effects on complex phenotypic traits. For example, DLK1 and neighboring MEG3 genes located in the region of the callipyge locus responsible for muscle hypertrophy in sheep [70] are interesting candidate genes for economically important traits. Various studies have shown their role in the regulation of adipogenesis, in muscle development and fetal growth [71–73]. Similarly, IGF2 plays an essential role in growth and differentiation. These genes are associated with QTLs for growth and fattening and consequently in meat quality-related traits [25, 74]. Determining whether the imprinted gene network is conserved between species and the role of new factors (multigene complexes, lncRNAs, microRNAs) in gene interactions will help to understand mechanisms associated with imprinted genes, but also may have implications for future animal breeding research [75, 76].

## Additional files

Additional file 1: Table S1. Information on the genes studied and mapping results in pig. (DOCX 16 kb)
Additional file 2: Figure S1. a, Distribution of DNA spot centers to CT edge distances for both alleles located outside of CT is shown. For both alleles, the maximal distance found between RNA spot center and CT edge reached 2 μm. No statistically significant difference was found between the two alleles; however the distribution shows a tendency of the expressed allele to be more frequently outside the CT; b, Heat map representing the position of pair of alleles in each nucleus. The position of each allele towards its CT is analyzed within the 9 different combinations of position. No statistically significant difference was found (*n* = 51 nuclei). (PPTX 84 kb)
Additional file 3: Figure S2. IGF2 - (DLK1-MEG3) DNA positioning relative to CTs (SSC2 and SSC7) in liver cells. Two examples (a, b) of decomposed confocal images showing 3D DNA FISH of IGF2 and DLK1/MEG3 loci in an undifferentiated color (white) (I) and merge (II) with specific chromosome painting probes (SSC2 in red and SSC7 in green). White arrows on merge images indicate the position of proximal alleles. The nucleus is counterstained with DAPI (blue). (PPTX 540 kb)
Additional file 4: Figure S3. Analysis of the interaction between IGF2-DLK1 and IGF2-MEG3 RNAs in muscle cells. Decomposed confocal images and merge showing RNA hybridization signals for: a, I: IGF2 (red), II, DLK1 (green) RNAs and III merge; b, I: MEG3 (red), II: IGF2 (green) RNAs and III merge. White arrows on merge images indicate colocated RNAs. The nucleus is counterstained with DAPI (blue). (PPTX 636 kb)

## Abbreviations

2D FISH: Two-dimensional fluorescence *in situ* hybridization
3D FISH: Three-dimensional fluorescence *in situ* hybridization
BAC: Bacterial artificial chromosome
CT: Chromosome territory
DAPI: 4′, 6′ Diamidino-2-phenylindole
SSC: Sus scrofa domestica
TSA: Tyramide Signal Amplification
