# Supplementary material for: Expressed alleles of imprinted IGF2, DLK1 and MEG3 colocalize in 3D-preserved nuclei of porcine fetal cells
Source: BMC Cell Biol. 2016 Oct 1;17:35. doi: 10.1186/s12860-016-0113-9 (PMC5045652; doi:10.1186/s12860-016-0113-9)
Supplement: Additional file 1: Table S1. — Information on the genes studied and mapping results in pig. (DOCX 16 kb) [file 12860_2016_113_MOESM1_ESM.docx]

| **Gene Symbol (alias)** | **Full name** | **Imprinting Status** | **Expressed allele in Human** | **Present in IGN*** | **Porcine sequence** | **BAC number** | **Specific primers** | **Size PCR products** | **Localization in pig** |
| --- | --- | --- | --- | --- | --- | --- | --- | --- | --- |
| IGF2 | Insulin growth factor 2 | imprinted | Pat | yes | NM_213883 | PigI-370D12 | GCATCGTGGAAGAGTGCTG  TCCAGGTGTCATAGCGGAAG | 458 bp | SSC 2p17 |
| SLC38A4 | Solute carrier family 38, member 4 | imprinted | Pat | yes | AK232278 | PigI-759D10 | CTGAAGGAGCTTGTGTGTGG  CTGCTCATTGCTGCCTTTTC | 162 bp | SSC 5q21-23 |
| ZAC1 (PLAGL1) | Zinc finger protein which regulates apoptosis and cell cycle arrest | imprinted | Pat | yes | EH009042 | PigI- 343G10 | ACAACACCATGCTGGGCTAC  AGAAGCATCTCTCGCAGTGG | 198 bp | SSC 1pter |
| DLK1 | Delta-like 1 homolog (Drosophila) | imprinted | Pat | yes | EW669278 | PigI-790B6 | GGCATCGTCTTCCTCAACAA  CGCTGCTTAGATCTCCTC | 194 bp | SSC 7q26 |
| MEG3 (GTL2) | Maternally expressed 3 (non-protein coding) | imprinted | Mat | yes | EF468461 | PigI-790B6 | ACCAGCCTACGAAGAAAGCC  GGAGAATAAATGAGACGGTGAG | 644 bp | SSC 7q26 |
| SEP15 | Selenoprotein 15 | No imprinted |  | no | EF178474 | CH242-178E23 | ACACAGACAGCGTGGAAGAA  CCTTACCTACCCTGGGCTCT | 208 bp | SSC 4qter |
| ZAR1 | Zygote arrest 1 | No imprinted |  | no | NM_001129956 | PigI-427B5 | CCTGTTTCCTCCTCTGACGG  CCACGTCTCGAATGCTGACT | 169 bp | SSC 8q11-12 |
| NF1 | Neurofibromin 1 | No imprinted |  | no | AJ943899 | PigI-0880B10 | CCCTGCAAGAATGGATCAAC  TTGCCCTAGGTGTTCAGAGC | 826 bp | SSC 12q |
| OSBP2 | Oxysterol binding protein 2 | No imprinted |  | no |  | PigI-576 E10 | TTGAGCTTCCTCCAACGACTC  CCCAGAAGTGGGTTGAAAGG | 1479 bp | SSC 14q21 |
| OSBPL1 | Oxysterol binding protein-like 1A | No imprinted |  | no | AK233522 | PigI-019D10 | CGTTCTCCTGTGGCGAATAG  CGCTCTGATGTCAGGTCGTAG | 527 bp | SSC 6q27-28 |
| ABCG2 | ATP-binding cassette, sub-family G (WHITE), member 2 | No imprinted |  | no | AJ420927 | PigI-1035G10 | AGTGTTTCAGCAGCGTCTCCG  TGCTTGGTAACATCCTCATGG | 1931 bp | SSC 8q26-27 |
| RPL32 | Ribosomal protein L32 | No imprinted |  | yes | EW027713 | EW027713 | TCATACTGTGCTGAGATTGCTC  CTGGCATTGGGATTGGTG | 104 bp | SSC 13q24-33 |
| GPC3 | Glypican 3 | No imprinted |  | no | DT332132 | PigI-910H5 | CCGAATGTGGTATTGCTCGT  ACTGGATGGAGTCGTGGATG | 227 bp | SSC Xq26 |

* IGN: Imprinting Gene Network

**Additional File 1: Table S1**: Information about genes studied and mapping results in pig
