# Supplementary material for: Expressed alleles of imprinted IGF2, DLK1 and MEG3 colocalize in 3D-preserved nuclei of porcine fetal cells
Source: BMC Cell Biol. 2016 Oct 1;17:35. doi: 10.1186/s12860-016-0113-9 (PMC5045652; doi:10.1186/s12860-016-0113-9)
Supplement: Additional file 2: Figure S1. — a, Distribution of DNA spot centers to CT edge distances for both alleles located outside of CT is shown. For both alleles, the maximal distance found between RNA spot center and CT edge reached 2 μm. No statistically significant difference was found between the two alleles; however the distribution shows a tendency of the expressed allele to be more frequently outside the CT; b, Heat map representing the position of pair of alleles in each nucleus. The position of each allele towards its CT is analyzed within the 9 different combinations of position. No statistically significant difference was found (n = 51 nuclei). (PPTX 84 kb) [file 12860_2016_113_MOESM2_ESM.pptx]

## Slide 1
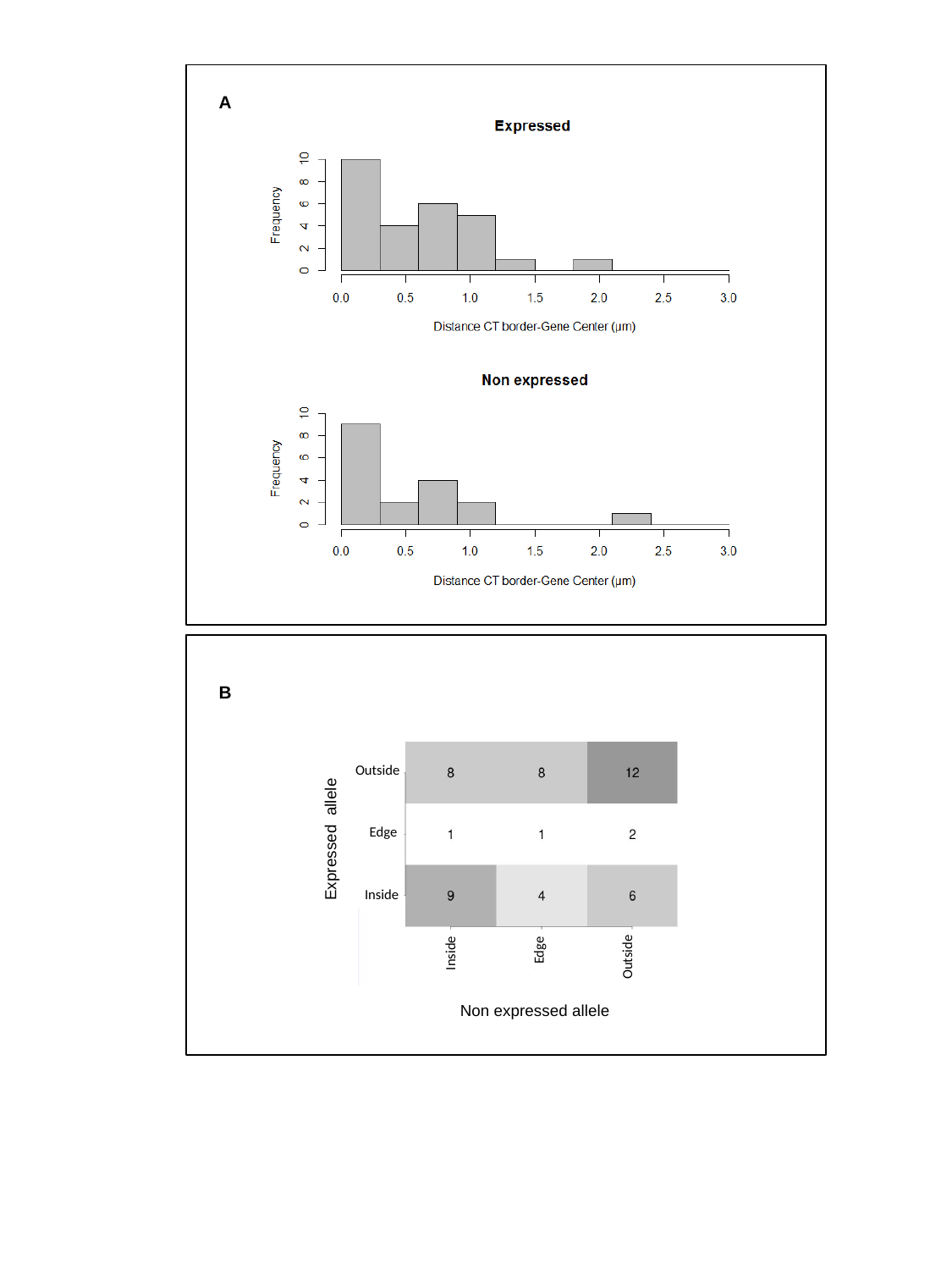

A
B
Non expressed allele
Expressed allele
Outside
Edge
Inside
Outside
Edge
Inside
Outside
