# Supplementary figures and images for: Expressed alleles of imprinted IGF2, DLK1 and MEG3 colocalize in 3D-preserved nuclei of porcine fetal cells
Source: BMC Cell Biol. 2016 Oct 1;17:35. doi: 10.1186/s12860-016-0113-9 (PMC5045652; doi:10.1186/s12860-016-0113-9)

## Slide 1
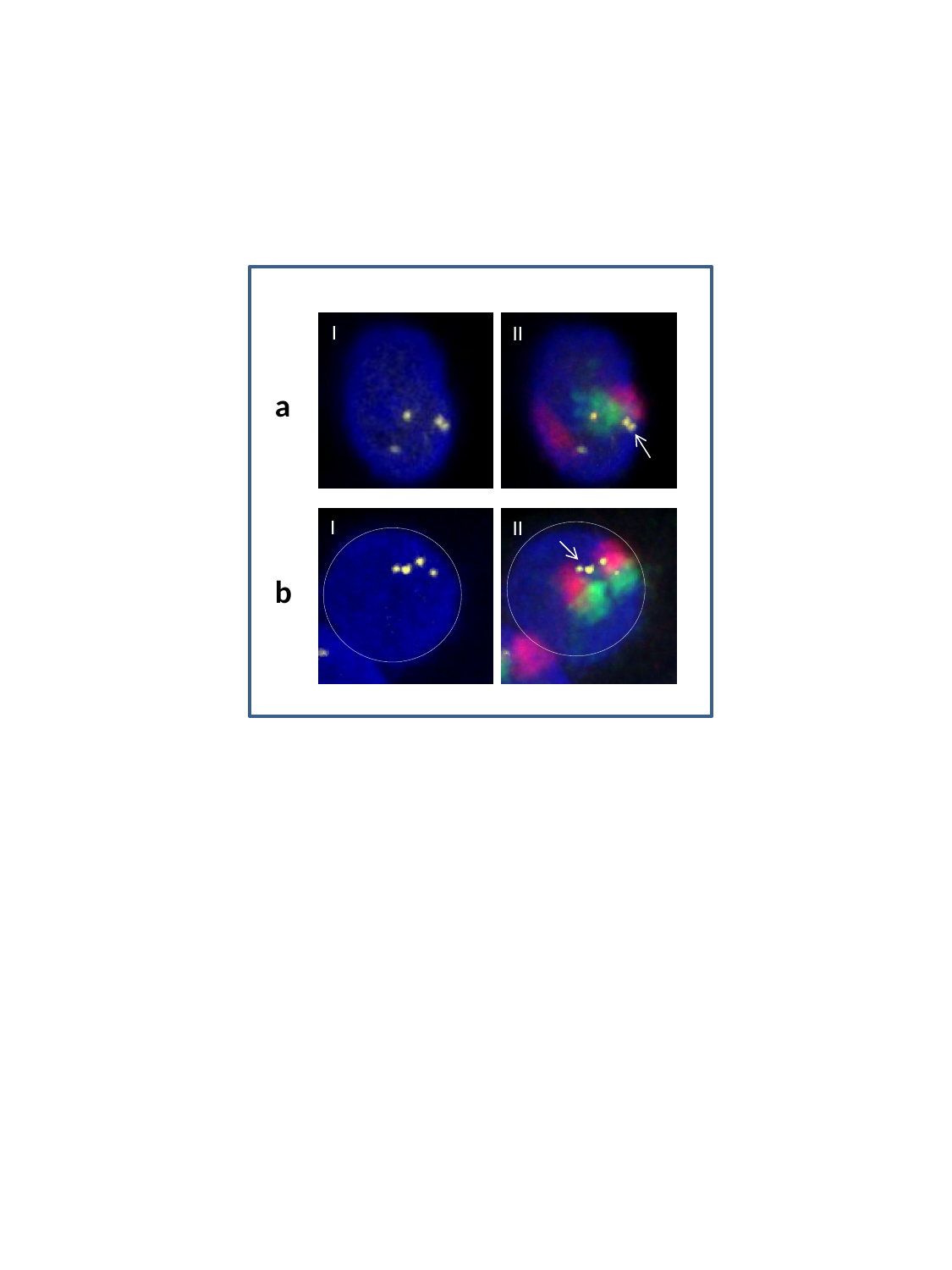

I
II
a
b
I
II
I
II

Supplement: Additional file 3: Figure S2. — IGF2 - (DLK1-MEG3) DNA positioning relative to CTs (SSC2 and SSC7) in liver cells. Two examples (a, b) of decomposed confocal images showing 3D DNA FISH of IGF2 and DLK1/MEG3 loci in an undifferentiated color (white) (I) and merge (II) with specific chromosome painting probes (SSC2 in red and SSC7 in green). White arrows on merge images indicate the position of proximal alleles. The nucleus is counterstained with DAPI (blue). (PPTX 540 kb) [file 12860_2016_113_MOESM3_ESM.pptx]

## Slide 1
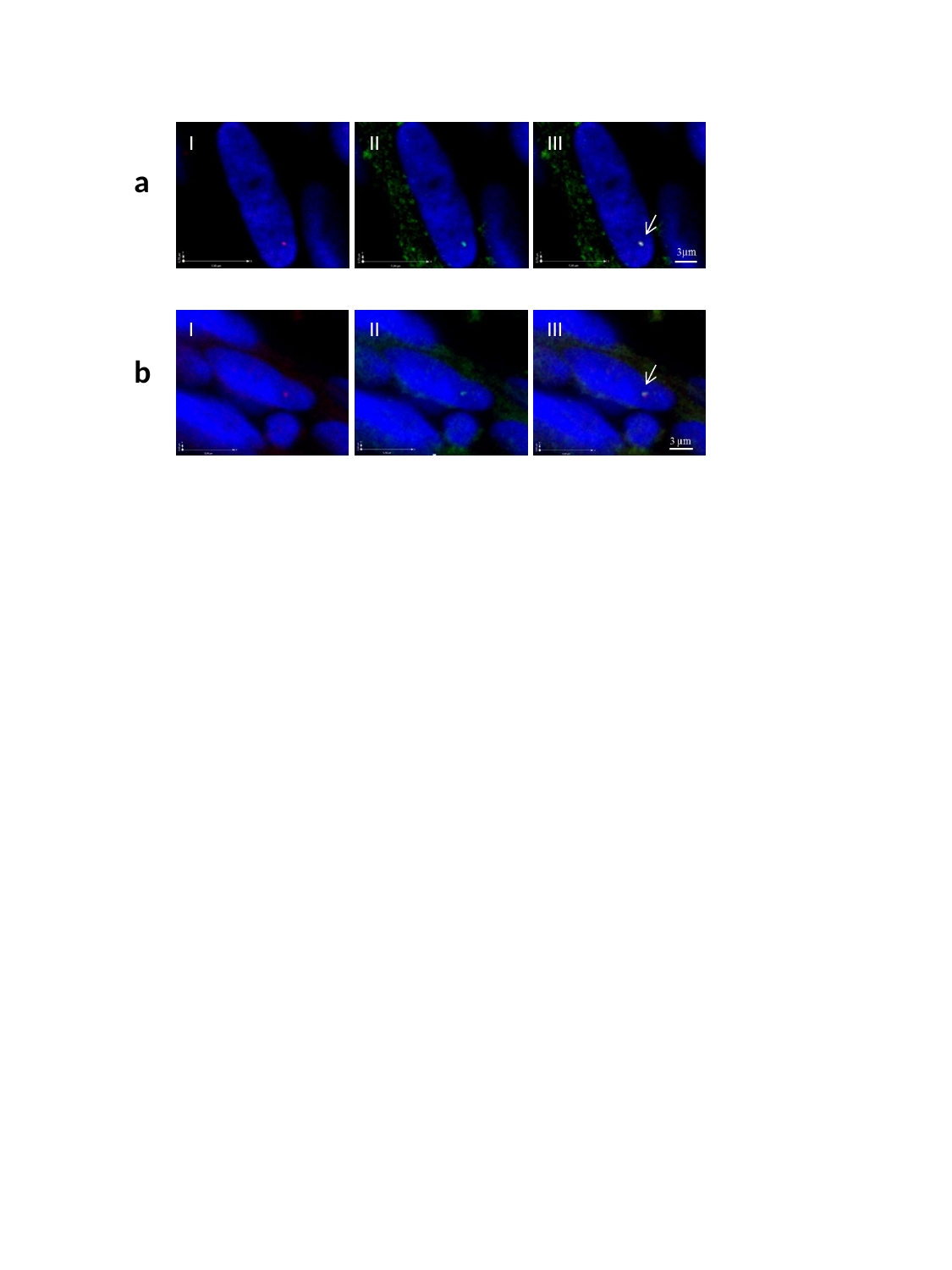

I
II
III
a
I
II
III
b

Supplement: Additional file 4: Figure S3. — Analysis of the interaction between IGF2-DLK1 and IGF2-MEG3 RNAs in muscle cells. Decomposed confocal images and merge showing RNA hybridization signals for: a, I: IGF2 (red), II, DLK1 (green) RNAs and III merge; b, I: MEG3 (red), II: IGF2 (green) RNAs and III merge. White arrows on merge images indicate colocated RNAs. The nucleus is counterstained with DAPI (blue). (PPTX 636 kb) [file 12860_2016_113_MOESM4_ESM.pptx]
